# Proteomic Identification of Small Extracellular Vesicle Proteins LAMB1 and Histone H4 for Prostate Cancer Diagnosis and Risk Stratification

**DOI:** 10.1002/advs.202402509

**Published:** 2024-04-08

**Authors:** Bairen Pang, Qi Wang, Haotian Chen, Zhihan Liu, Meng Han, Jie Gong, Liang Yue, Xuan Ding, Suying Wang, Zejun Yan, Yingzhi Chen, David Malouf, Joseph Bucci, Tiannan Guo, Cheng Zhou, Junhui Jiang, Yong Li

**Affiliations:** ^1^ Department of Urology The First Affiliated Hospital of Ningbo University Ningbo Zhejiang 315010 China; ^2^ Ningbo Clinical Research Centre for Urological Disease The First Affiliated Hospital of Ningbo University Ningbo Zhejiang 315010 China; ^3^ Translational Research Laboratory for Urology The Key Laboratory of Ningbo The First Affiliated Hospital of Ningbo University Ningbo Zhejiang 315010 China; ^4^ Zhejiang Engineering Research Center of Innovative technologies and diagnostic and therapeutic equipment for urinary system diseases Ningbo Zhejiang 315010 China; ^5^ Cancer Care Centre St George Hospital Kogarah NSW 2217 Australia; ^6^ St. George and Sutherland Clinical Campuses School of Clinical Medicine UNSW Sydney Kensington NSW 2052 Australia; ^7^ Health Science Centre Ningbo University Ningbo Zhejiang 315211 China; ^8^ Westlake Centre for Intelligent Proteomics Westlake Laboratory of Life Sciences and Biomedicine Hangzhou Zhejiang 310030 China; ^9^ Key Laboratory of Structural Biology of Zhejiang Province School of Life Sciences Westlake University Hangzhou Zhejiang 310030 China; ^10^ Department of Pathology Ningbo Diagnostic Pathology Centre Ningbo Zhejiang 315021 China; ^11^ Department of Urology St George Hospital Kogarah NSW 2217 Australia

**Keywords:** biomarker, diagnosis, extracellular vesicle, Histone H4, LAMB1, prostate cancer

## Abstract

Diagnosis and stratification of prostate cancer (PCa) patients using the prostate‐specific antigen (PSA) test is challenging. Extracellular vesicles (EVs), as a new star of liquid biopsy, has attracted interest to complement inaccurate PSA screening and invasiveness of tissue biopsy. In this study, a panel of potential small EV (sEV) protein biomarkers is identified from PCa cell lines using label‐free LC‐MS/MS proteomics. These biomarkers underwent further validation with plasma and urine samples from different PCa stages through parallel reaction monitoring‐based targeted proteomics, western blotting, and ELISA. Additionally, a tissue microarray containing cancerous and noncancerous tissues is screened to provide additional evidence of selected sEV proteins associated with cancer origin. Results indicate that sEV protein LAMB1 is highly expressed in human plasma of metastatic PCa patients compared with localised PCa patients and control subjects, while sEV protein Histone H4 is highly expressed in human urine of high‐risk PCa patients compared to low‐risk PCa patients and control subjects. These two sEV proteins demonstrate higher specificity and sensitivity than the PSA test and show promise for metastatic PCa diagnosis, progression monitoring, and risk stratification.

## Graphical abstract

Novel biomarkers with better specificity and sensitivity facilitate PSA in PCa diagnosis and treatment decision making. This study discovers two critical sEV biomarkers, LAMB1 and Histone H4, which also validated in several independent cohorts with different specimens (plasma, urine, tissue) showing a higher specificity and sensitivity than PSA test. These findings propose sEV LAMB1 and Histone H4 as novel biomarkers hold promise for PCa diagnose and risk progression stratification.

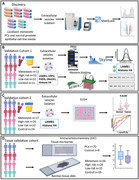

## 1 Introduction

Prostate cancer (PCa) is the second most common cancer in men and nearly one and a half million men are diagnosed annually world‐wide.^[^ ^1^ ^]^ More alarming still, the percentage of patients with metastatic disease at diagnosis has increased by 25% over the last decade.^[^ ^2^ ^]^ Several studies indicate that detecting PCa at an early stage reduces mortality.^[^ ^3^ ^]^ Despite surgery and radiation therapy that can treat localised disease, up to 30% of treated PCa patients suffer relapse and metastasis. There is no curative therapy for metastatic PCa (mPCa) and median survival is ≈1 year. The survival of PCa patients is largely related to tumour stage and grade.^[^ ^4^ ^]^ Treatment options for different stages of PCa patients vary with risk grade.

A major clinical challenge in PCa management is posed by the inability of current diagnostic tests, such as serum prostate‐specific antigen (PSA) testing, digital rectal examination, and histopathologic grading of tissues, to discern between indolent and aggressive disease.^[^ ^5^ ^]^ A major knowledge gap for PCa research is the lack of suitable biomarkers for accurate diagnosis, therapeutic stratification, and predicting or monitoring metastasis. The benefits of risk stratification are to assess the overall progression of metastases for localised cancer, select optimal treatment options, and predict the likelihood of recurrence following definitive local therapy. Therefore, improving the screening and early diagnosis of PCa, especially metastatic disease, are of great importance in designing reasonable treatment plans and improving the prognosis of patients.

The current gold standard for PCa diagnosis and stratification, the serum PSA test, is both invasive and inaccurate. It has been reported that a PSA cutoff at 2.0 ng mL^−1^ misses more than 45% of biopsy‐detectable PCa.^[^ ^6^ ^]^ Data also suggests that a large number of men with a PSA score <4 ng mL^−1^ may have undetectable PCa, while as many as 75% of men test positive for PSA (>4.0 ng mL^−1^) without PCa.^[^ ^7^ ^]^ The low specificity and sensitivity of PSA typically results in 20% to 42% overdiagnosis and overtreatment, suggesting more harm than good for patients.^[^ ^4^, ^8^ ^]^ PSA screening and derived scoring systems (including PHI and 4Kscore) lack specificity and accuracy, and subsequently result in overdiagnosis with the possibility of being accompanied with pain, bleeding, sexual dysfunction, urgency of urination, acute urinary retention, or even life‐threatening septicaemia in up to 2% of cases.^[^ ^9^ ^]^ In addition, magnetic resonance imaging has improved diagnostic selection to some extent, but utility is still limited by 5–15% false negatives and 29–59% false positives using Prostate Imaging Reporting and Data Systems scoring.^[^ ^10^ ^]^

Extracellular vesicles (EVs) are derived from parental cells and secreted into biofluids. EVs are a general term and include different subpopulations such as small EVs (sEVs) and large EVs (lEVs). sEVs are the most investigated EV subpopulation as they are rich in biological cargo information including nucleic acids, metabolites, and proteins and play an important role in intercellular cancer communication and metastasis. Accumulating evidence has demonstrated that tumour‐derived sEV proteins are a valuable source of biomarkers from liquid biopsy for cancer diagnosis, monitoring, and prognosis.^[^ ^11^ ^]^

Mass spectrometry (MS)‐based proteomic analysis is an efficient and widely used tool to characterise sEV proteins. Recently, a multiple human cancer proteomic profiling of sEVs from 426 human samples showed that a panel of sEV proteins from tissue explants and plasma had the ability to classify tumours from unknown primary origin.^[^ ^11a^ ^]^ However, sEVs isolated from biofluids can consist of heterogeneous populations. Therefore, quantitative proteomic strategies alone may fail to establish the correct localisation and orientation of sEV proteins due to an insufficient isolation strategy and protein abundance variation. Therefore, further validation experiments are often required, such as parallel reaction monitoring (PRM), western blot (WB), and enzyme‐linked immunosorbent assay (ELISA) to overcome MS‐based interference and validate the specificity of potential EV protein biomarkers for clinical translation.

In this study, liquid chromatography with tandem MS (LC‐MS/MS)‐based label‐free quantitative proteomics was used to identify novel proteins associated with PCa. Potential biomarkers were screened with PCa cell lines and verified in human plasma and urine samples using PRM, WB and ELISA.

## 2 Results

### 2.1 Study Design and Characterisation of sEVs from different Sample Origins

**Figure 1:**
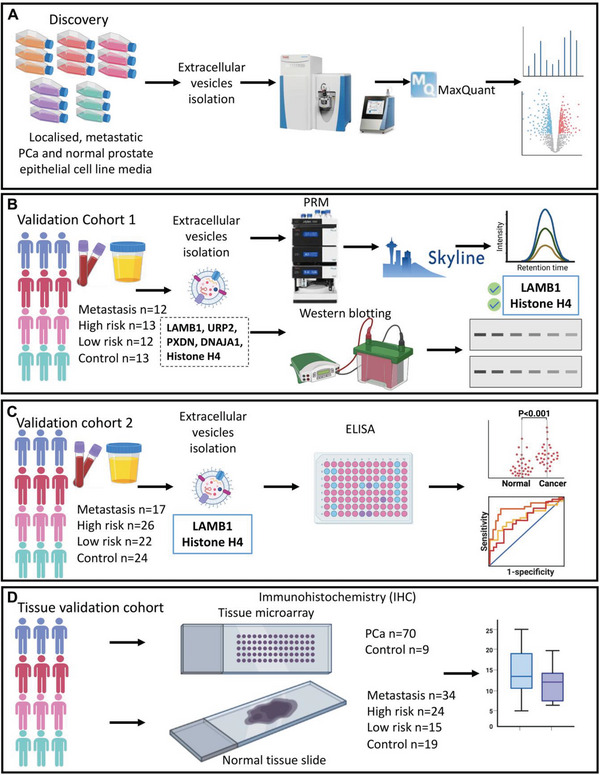
Schematic diagram of study design. A) The discovery of potential sEV biomarkers with label‐free LC‐MS/MS proteomic analysis using metastatic PCa cell lines (PC3, DU145, LNCaP), a primary PCa cell line (22Rv1), and a normal prostate epithelial cell line (RWPE‐1). B) Validation of sEV protein candidate biomarkers in human plasma and urine samples with PRM and WB, indicated that LAMB1 and Histone H4 were found to have significant differences among each group. C) Validation of sEV proteins LAMB1 and Histone H4 in a second independent cohort of plasma and urine samples from different stages of PCa patients using ELISA. D) An additional independent tissue validation cohort including PCa and normal prostate tissue from tissue microarrays and our tissue archive were performed to verify the expression levels of LAMB1 and Histone H4 proteins in PCa tissue. n represent for total participants in each group. Abbreviations. ELISA: enzyme‐linked immunosorbent assay; LC‐MS/MS: liquid chromatography with tandem mass spectrometry; PRM: parallel reaction monitoring; PCa: prostate cancer; sEVs: small extracellular vesicle; WB, western blot.

For the discovery of potential PCa‐derived biomarkers, label‐free quantitative proteomic analysis of sEV samples from 4 different human PCa cell lines and one human normal prostate epithelial cell line was performed. From this analysis, several groups of differentially expressed proteins in primary PCa and various mPCa cell groups were identified (**Figure** **1**A). Further verification of these potential biomarkers was conducted by targeted proteomics (PRM) on sEV plasma and urine samples isolated from an independent cohort of PCa patients (Figure 1B).

Results showed that the plasma sEV protein LAMB1 had significantly higher expression levels in the metastatic group of PCa patients compared to the high‐risk and control groups [healthy and benign prostatic hyperplasia (BPH) participants]. Similarly, the verification of potential biomarkers selected from PCa urine samples showed that sEV protein Histone H4 had higher expression levels in the high‐risk group compared to the low‐risk and control groups (Figure 1B). Relatively higher expression of sEV proteins LAMB1 and Histone H4 was confirmed by WB (Figure 1B). To investigate the potential of clinical translation, the sEV proteins LAMB1 and Histone H4 were analysed using ELISA on the second independent cohort of PCa plasma and urine samples (Figure 1C). We observed significantly higher expression of the plasma sEV protein LAMB1 in the metastatic group and the overall PCa group. Additionally, the expression of the urine sEV protein Histone H4 was higher in the high‐risk group. More importantly, both LAMB1 and Histone H4 demonstrated higher diagnostic values than PSA in their respective Receiver Operating Characteristic (ROC) curves (Figure 1C). To further investigate the association of sEV proteins LAMB1 and Histone H4 with human PCa tissue, the verification of the LAMB1 and Histone H4 protein expression on tissue samples were applied using commercial tissue microarray (TMA) available and paraffin‐embedded tissue slides from our tissue archive in another cohort (Figure 1D). Elevated levels of LAMB1 were observed with the development of PCa, but no significant increase in Histone H4 levels was observed when comparing high‐risk to low‐risk PCa (Figure 1D).

**Figure 2:**
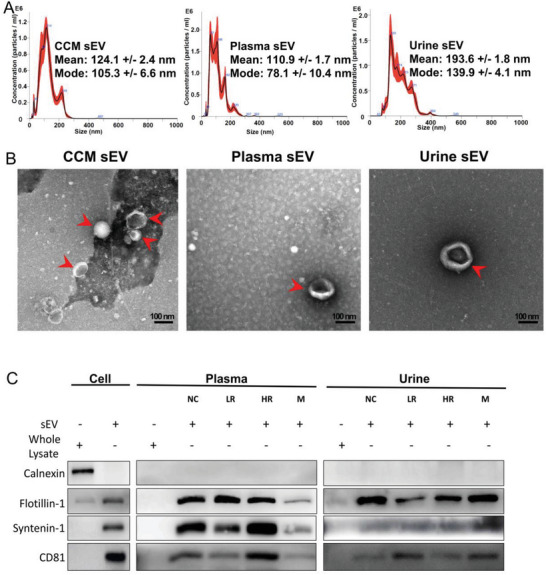
Characterisation of sEVs isolated from PCa cell lines, and human PCa plasma and urine samples. A) Particle size distributions of sEV preparations from CCM, plasma, and urine were measured by nanoparticle tracking analysis. B) Representative transmission electron microscopy images of sEV (*red arrows*) isolated with a lipid bilayer and cup‐shaped structure. Scale bar‐100 nm. C) Representative western blots showing expression of flotillin‐1, syntenin‐1, CD81, and calnexin for sEV preparations separated from CCM, plasma, and urine compared with whole cell lysate, whole plasma lysate, and whole urine lysate. Whole cell lysate from PCa PC3 cells was used to compare against sEVs derived from CCM of the PC3 cell line, human plasma, and urine. 2.5 µg of protein from whole cell lysate and CCM sEV preparation was loaded; 4.2 µg of protein from whole plasma lysate and plasma sEV preparation was loaded; and 1.7 µg of protein from whole urine lysate and urine sEV preparation was loaded. Abbreviations. CCM: cell culture medium; HR: high‐risk; LR: low‐risk; M: metastasis; NC: control; PCa: prostate cancer; sEV: small extracellular vesicle.

To characterise sEVs isolated from cell culture medium (CCM), plasma, and urine samples, nanoparticle tracking analysis (NTA) was used to assess particle size and distribution. Particles from different biofluids were found to be within the size range of sEVs (**Figure** **2**A). Particle modal sizes were 105.3 ± 6.6 nm for CCM, 78.1 ± 10.4 nm for plasma, and 139.9 ± 4.1 nm for urine; and particle mean sizes were 124.1 ± 2.4 nm for CCM, 110.9 ± 1.7 nm for plasma, and 193.6 ± 1.8 nm for urine. In addition, transmission electron microscopy (TEM) was used to observe the morphology of sEVs from different sample sources. Representative TEM images of sEV isolated from CCM, plasma, and urine samples are shown in Figure 2B. Clear cup‐shaped and lipid bilayer structures of sEVs were observed (Figure 2B), which were in accordance with our previous report.^[^ ^12^ ^]^ The cup shape indicates an intact bilipid membranous vesicle, but dehydrated and, therefore, not perfectly spherical. Non‐EV particles (i.e., lipids and protein aggregates) were also observed. The unclear background in the images may be due to the crystallisation of salt in the buffer.

Protein expression of sEV samples from different biofluids were also evaluated by WB. sEV markers including CD81, syntenin‐1, and flotillin‐1 were used to confirm the presence of sEVs isolated from CCM, plasma, and urine samples, respectively (Figure 2C). sEV samples isolated from plasma and urine with different clinical groups including control, low‐risk, high‐risk, and metastatic mPCa were all positive for these sEV markers. Calnexin was negative in all sEV preparations tested, indicating minimal cell lysate contamination and a high concentration of pure sEVs in this study.

### 2.2 Identification of Potential sEV Protein Biomarkers from PCa Cell Lines using LC‐MS/MS

To investigate protein biomarkers for PCa diagnosis and risk progression stratification, sEVs derived from 4 different PCa cell lines (PC3, DU145, LNCaP, 22Rv1) and a normal prostate epithelial cell line (RWPE‐1) were analysed by LC‐MS/MS label‐free proteomics. The results showed the identification of 1570 proteins and 878 of these were quantified (Figure S1A; Table S4, Supporting Information). Among them, there were 384 sEV proteins expressed in the RWPE‐1 cell line, 473 in PC3 cells, 480 in DU145 cells, 597 in LNCaP cells, and 570 in 22Rv1 cells (Figure S1B, Supporting Information). Furthermore, the principal component analysis of these qualitative comparisons highlighted the distinct protein composition of the normal RWPE‐1 sEV fraction compared to the PCa sEV fraction (Figure S1C, Supporting Information).

**Figure 3:**
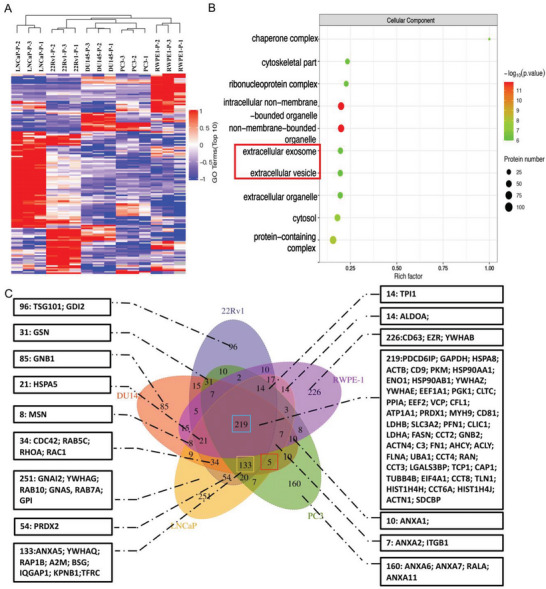
Analysis of sEV proteins from PCa cell lines. A) Heatmap shows the sEV proteins differentially expressed in PCa cell lines and a normal prostate epithelial cell line (RWPE‐1). B) Gene ontology enrichment analysis shows the top 10 sEV‐associated bioactivities in 5 different cell lines. Rich factor represents the proportion of differentially expressed proteins annotated to a certain structural domain category compared to all identified proteins annotated to that category. The vertical axis represents the statistical results of differential proteins under each structural domain classification. C) Venn diagram showing the detected sEV proteins from 5 different cell sources. Boxes highlight the key sEV protein markers identified in PCa cells. The results demonstrate the number of proteins identified in PCa cell lines (PC3, DU145, LNCaP, 22Rv1) in comparison with a normal prostate epithelial cell line (RWPE‐1). FDR <0.01 and unique peptides ≥2 for mass spectrometry data analysis. Overlapping sections show the number of shared proteins, while the side sections show the uniquely expressed proteins. Each cell line is depicted with a unique colour. Abbreviations. PCa: prostate cancer; sEV: small extracellular vesicle.

Proteomic profiling of sEVs in PCa cell lines is shown in **Figure** **3**. Unsupervised hierarchical clustering showed a clear separation of different PCa cell lines and one normal prostate epithelial cell line based on sEV proteins (Figure 3A). Gene ontology (GO) enrichment analysis of the cellular component showed that “extracellular exosome” and “extracellular vesicle” were enriched in all 5 unique cell line‐derived sEV samples when comparing all differentially expressed sEV samples together (Figure 3B). This finding underscores the significant role that sEVs and their carried cargo proteins play in PCa. Consequently, to delve deeper into the protein content of sEVs for the subsequent study of differentially expressed protein biomarkers and further explore the associations among sEVs from different cell lines, a Venn diagram was performed to illustrate the unique proteins and protein overlaps (Figure 3C). 219 shared proteins (LAMB1, Histone H4, est.) were identified in sEV samples obtained from all 5 cell lines, 133 sEV proteins (URP2, DNAJA1, est.) were expressed exclusively in PCa cell lines (PC3, DU145, LNCaP, 22Rv1), and 5 sEV proteins (RTN4, LARS1, est.) were expressed exclusively in mPCa cell lines (PC3, DU145, LNCaP). Several previously reported PCa biomarkers, such as GPC1, CD44, PSA, and PSMA, were also detected in the PCa‐secreted sEVs.^[^ ^13^ ^]^ EV markers from the Vesiclepedia database were investigated (Figure 3C boxes) to illustrate the accuracy of this sEV study and provide a detailed distribution of these proteins in the sEVs from PCa cell lines.^[^ ^11^, ^14^ ^]^ For example, traditional tetraspanins such as CD9 and CD81 were found in sEVs from all 5 cell lines.

Although a large number of sEV proteins were identified from all cell lines, each cell type exhibited enrichment of a unique set of proteins. Among the sEV proteins discovered, we searched for sEV proteins identified as unique in mPCa cell lines or unique in metastatic and localised PCa cell lines, or with significantly differential protein expression level in mPCa cell lines compared to localised PCa or normal prostate epithelial cell line. Results indicate that most identified proteins are expressed in both PCa cells and normal cells, suggesting the difficulty in identifying an EV cancer protein marker that is totally negative in normal cells, which is reasonable based on EV communication properties between normal cells and cancer cells. Therefore, focus was placed on sEV protein expression differences between PCa cells and normal cells, based on a previous similar cancer biomarker study.^[^ ^15^ ^]^

To identify proteins found at high frequency in the tested samples, proteins with medium or high feasibility present in >50% of the PCa samples (among three replicates, proteins were quantified in at least two of the replicates) were searched, and, of those, 20 proteins (**Table** **1**) were selected for further validation using plasma and urine samples in PCa patients at different stages, according to the results in comparisons of interesting groups between mPCa cell lines, localised primary PCa cell line, normal prostate epithelial cell line. The selection of 20 biomarkers present in >50% PCa samples was based on a previous publication.^[^ ^11a^ ^]^ The criterion of “present in >50%” indicates that, among three replicates, proteins were quantified in at least two of the replicates. The potential PCa sEV protein markers including RTN4, LARS1, LAMB1, LAMB2, PXDN, NRP1, ABI3BP, FLNC, and SPON2 are highly expressed in sEVs from mPCa cell lines, but absent or minimally expressed in sEVs from a localised primary PCa cell line or a normal prostate epithelial cell line; URP2, DNAJA1, ADK, Histone H4, CLTC, AHCY, MYH9, Histone H3.1, RAN, FLNA, and CD151 are highly expressed in sEVs from PCa cell lines, but absent or minimally expressed in sEVs from normal prostate epithelial cells. These findings indicate that differential expression of sEV protein biomarkers can be identified using proteomics for further clinical validation and translation.

**Table 1:** Twenty potential sEV protein markers identified from PCa cell lines.

Protein name	Feasibility	sEV intensity ratio	Cancer association
RTN4	high	Infinity ^a^ ^)^	Cancer metastasis
LARS1	high	Infinity ^a^ ^)^	
LAMB2	high	Infinity ^a^ ^)^	
PXDN	high	58.5980613 ^a^ ^)^	
NRP1	high	16.4008783 ^a^ ^)^	
LAMB1	high	2.46038878 ^b^ ^)^	
URP2	high	Infinity ^c^ ^)^	Cancer development and progression
ADK	high	Infinity ^c^ ^)^	
DNAJA1	high	Infinity ^c^ ^)^	
AHCY	high	44.2794228 ^c^ ^)^	
MYH9	high	18.5861744 ^c^ ^)^	
Histone H3.1	medium	9.0234375 ^c^ ^)^	
CLTC	high	6.76419375 ^c^ ^)^	
Histone H4	high	6.06402446 ^c^ ^)^	
RAN	high	5.54583894 ^c^ ^)^	
FLNA	high	1.93085254 ^c^ ^)^	
CD151	medium	Infinity ^c^ ^)^	
ABI3BP	high	Infinity ^d^ ^)^	Bone metastasis
FLNC	high	Infinity ^e^ ^)^	Visceral metastases
SPON2	high	Infinity ^f^ ^)^	Lymph nodes metastases

Notes: Cancer association information of sEV proteins is based on proteomic results of protein intensity on each cell line.

^a)^ metastatic PCa cell lines compared to normal prostate cell line;

^b)^ metastatic PCa cell lines compared to localised primary PCa cell line;

^c)^ PCa cell lines compared to normal prostate cell line;

^d)^ bone metastatic PCa cell line compared to normal prostate cell line;

^e)^ brain metastatic PCa cell line compared to normal prostate cell line;

^f)^ lymph nodes metastatic PCa cell line compared to normal prostate cell line.

### 2.3 Verification of Potential sEV Proteins Identified in different Stages of PCa Patients using PRM

To validate the potential sEV biomarkers identified from PCa cell lines and determine whether these proteins could be used as diagnostic or risk stratification biomarkers for PCa patients, targeted MS analysis was performed to quantify proteins or peptides in samples from clinical subjects with different disease risks and stages. PRM quantified 18 of the 20 proteins described earlier. PCa patients with different cancer progression stages including low‐risk, high‐risk, and metastasis were evaluated with potential sEV proteins identified. The fragment total area reported by Skyline‐daily software (19.0.0.149, University of Washington, USA) was applied to quantify the sEVs in plasma and urine samples with different stages of PCa. Plasma samples from patients with low‐risk PCa (n = 12), high‐risk PCa (n = 12), mPCa (n = 11), and control subjects (n = 12) were collected to isolate sEVs for evaluating the protein cargo changes. Patient grouping information is shown in Table S1 (Supporting Information).

**Figure 4:**
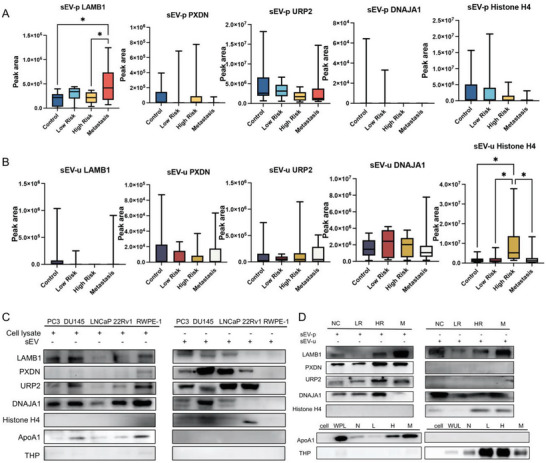
Verification of selected sEV proteins in PCa samples using PRM and WB. Expression levels of LAMB1, URP2, PXDN, DNAJA1, and Histone H4 protein in PCa cell lines and human PCa plasma and urine samples. A) Quantification of plasma sEV proteins LAMB1, PXDN, URP2, DNAJA1, and Histone H4 using targeted mass spectrometry. B) Quantification of urine sEV proteins LAMB1, PXDN, URP2, DNAJA1, and Histone H4 by targeted mass spectrometry. C) Expression of sEV proteins selected in PCa cell lines (PC3, DU145, LNCaP, and 22Rv1) in comparison with a normal prostate epithelial cell line (RWPE‐1) by WB. D) Expression of sEV proteins selected in plasma and urine samples with different stages of PCa patients by WB. Abbreviations. HR: high‐risk; LR: low‐risk; M: metastasis; NC: control; PCa: prostate cancer; PRM: parallel reaction monitoring; sEV: small extracellular vesicle; WB: western blotting.

Promisingly, results showed that sEV LAMB1 expression in plasma samples was found to be significantly altered between the high‐risk and mPCa groups (*P* = 0.0437) (**Figure** **4**A). In addition, sEVs derived from plasma samples also showed a significant difference between control and mPCa groups (*P* = 0.0227) (Figure 4A). LAMB1 expression in plasma sEVs was found to range from low to high as PCa progressed from the localised stage to metastasis. However, no significant difference was observed between the low‐risk and high‐risk groups for LAMB1 (*P*>0.05).

Similarly, sEV protein expression was evaluated using urine samples from different stages of PCa patients including low‐risk (n = 11), high‐risk (n = 12), metastatic (n = 11), and control subject (n = 12) groups. Expression of sEV protein Histone H4 in urine sEVs was found to be significantly increased in the high‐risk compared to the low‐risk (*P* = 0.0374), control (*P* = 0.0225), and metastatic (*P* = 0.0461) groups (Figure 4B). There was no significant difference found between the low‐risk and metastasis groups (*P* > 0.05). The peptide sequences used for LAMB1 and Histone H4 were YFQMSLEAEER and DAVTYTEHAK, respectively.

There was no significant difference found among other proteins tested such as PXND, URP2, and DNAJA1 in different stages of PCa in either plasma or urine samples (Figure 4A,B). To test the performance of the remaining selected sEV proteins, PRM analysis was also performed for LARS1, NRP1, LAMB2, MYH9 ADK, CLTC, RAN, Histone H3.1, FLNA, AHCY, FLNC, SPON2, and CD151 (Figures S2 and S3, Supporting Information). However, no significant difference was observed among the different clinical groups in both plasma and urine samples.

Our results indicate that the peptide profiles obtained through MS analysis show differences between plasma and urine sEV samples. Specifically, the sEV LAMB1 peptide in plasma samples and the sEV Histone H4 peptide in urine samples demonstrated significant results and therefore are candidates for further investigation. Furthermore, these results also further imply that, in addition to a small fraction of PCa‐derived proteins, sEV from patient‐derived biofluids may mix a large number of non‐PCa cell‐specific proteins, which may relate to the biofluids origin.

### 2.4 Verification of Potential sEV Proteins Identified in Human Plasma and Urine Samples using WB

To further examine the utility of sEV proteins selected from label‐free proteomic analysis, the expression of potential sEV protein markers were evaluated in PCa cell lines (PC3, DU145, LNCaP, 22Rv1) and a normal prostate epithelial cell line (RWPE‐1) using WB. Results indicate that the expression of sEV proteins LAMB1 and Histone H4 in PCa cell lines align with the results using label‐free proteomics (Figure 4C,D). That is, LAMB1 from mPCa cell lines and plasma samples of mPCa patients have the highest expression while Histone H4 from PCa cell lines and urine of PCa patients is elevated. However, expression of sEV proteins in normal RWPE‐1 cells and PCa cell lines have significant differences (Figure 4C). The reason for this observation may be the different mechanisms of autocrine or active secretion of sEVs to regulate nearby or distant cells by different cell types.^[^ ^16^ ^]^

Human plasma and urine samples from patients with different stages of PCa were analysed to further confirm our findings. However, WB could only partially verify results from LC‐MS/MS analysis (Figure 4C,D). Notably, the expression level of sEV proteins identified in plasma and urine exhibited significant differences (Figure 4), suggesting the variability in the expression of sEV protein biomarkers identified in PCa cell lines when tested in human clinical samples. Specifically, expression levels of sEV proteins LAMB1, PXDN, DNAJA1, and URP2 were found to be increased in plasma with PCa progression, but lower expression of DNAJA1 was found in the plasma sEVs of the metastasis group. Similarly, in urine‐derived sEVs, LAMB1, DNAJA1, and Histone H4 were found to be increased with PCa progression, but high levels of LAMB1 and DNAJA1 were also found in urine sEVs from the control group (Figure 4D). Apolipoprotein A1 (ApoA1) and Tamm‐Horsfall protein (THP), the major proteins with sEV isolation from plasma and urine, respectively, were used to indicate the origin of sEVs (Figure 4C,D). These findings are consistent with the PRM results (Figure 4A,B), providing additional evidence for further exploring the clinical diagnostic value of sEV proteins LAMB1 and Histone H4.

### 2.5 Validation of sEV Proteins LAMB1 and Histone H4 in Human Plasma and Urine by ELISA

To further validate the clinical value of sEV proteins LAMB1 and Histone H4 to develop a liquid biopsy‐based assay for the detection of mPCa and facilitate PCa stratification, the validation of the diagnostic performance of sEV proteins LAMB1 and Histone H4 in a real clinical setting is warranted. To this end, these sEV proteins were analysed in an independent set of plasma and urine samples from patients with different stages of PCa by ELISA, respectively, which is a test already implemented in daily clinical practice. Of note, expression levels of sEV proteins LAMB1 and Histone H4 measured in plasma and urine samples were highly consistent with the expression patterns observed by PRM (Figure 4C,D), further reinforcing the potential clinical utility of these two sEV proteins for the following translation from bench to bed.

**Figure 5:**
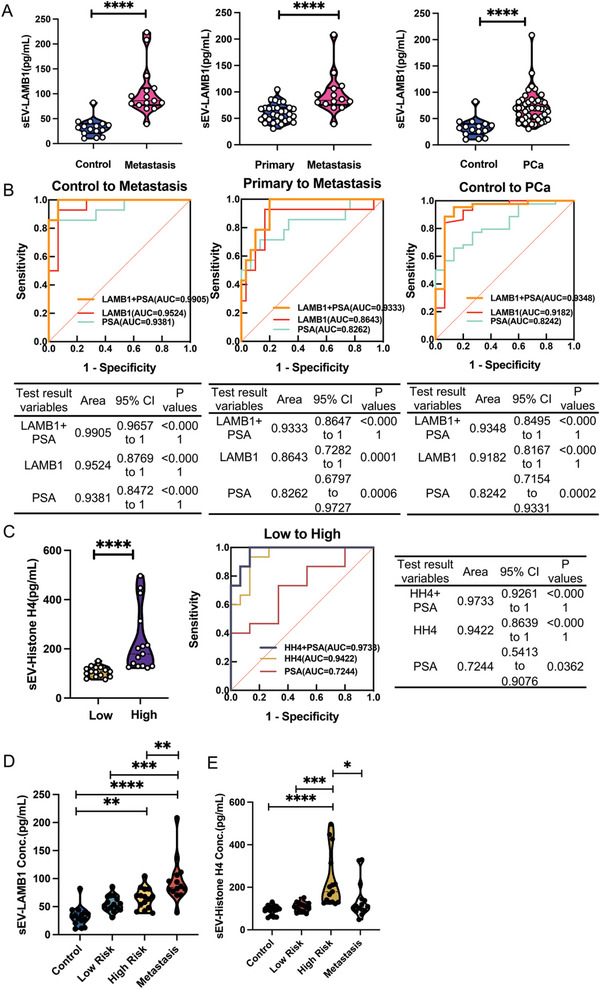
Clinical validation of sEV proteins LAMB1 and Histone H4 in human plasma and urine samples from PCa patients. A) Validation of sEV protein LAMB1 in clinical plasma samples with control (n = 15), localised primary PCa (n = 30), mPCa (n = 15), and total PCa (n = 45) groups. Metastasis PCa group vs. control group with *P*<0.0001; Metastasis group vs. primary PCa group with *P*<0.0001; PCa group vs. control group with *P*<0.0001. B) Comparison of receiver operating characteristic (ROC) curves in the diagnostic values for sensitivity and specificity using sEV protein LAMB1, serum PSA test, or combination of both by ELISA. C) Validation of sEV protein Histone H4 expression in clinical urine samples with low‐ (n = 15) and high‐risk (n = 15) PCa groups using ELISA and comparison of ROC curves in diagnostic values for sensitivity and specificity using sEV protein Histone H4, serum PSA test, or combination of both by ELISA. High‐risk PCa group vs. low‐risk PCa group *P*<0.0001. D, E) Comparison of sEV protein LAMB1 (n = 15 for control, low‐risk PCa, high‐risk PCa, and metastasis PCa groups) or sEV protein Histone H4 (n = 15 for control, low‐risk PCa, high‐risk PCa, and metastasis PCa groups) for PCa progression stratification. In plasma sEV protein LAMB1: Metastasis group vs. high‐risk group, *P* = 0.0062; Metastasis group vs. low‐risk group, *P* = 0.0002; Metastasis group vs. control group, *P<*0.0001; High‐risk group vs. control group, *P* = 0.0031. In urine sEV protein Histone H4: High‐risk group vs. control group, *P*<0.0001; High‐risk group vs. low‐risk group, *P* = 0.0005; Metastasis group vs. high‐risk group, *P* = 0.0245; PCa represent primary and metastatic together.**: P<0.01, ***: P<0.001, ****: P<0.0001. Abbreviations. PCa: prostate cancer; sEV: small extracellular vesicle.

Furthermore, LAMB1 was significantly increased in patients with mPCa compared to controls (healthy and BPH participants) (*P*<0.0001) and localised primary PCa patients (*P*<0.0001) (**Figure** **5**A). The increased accuracy for mPCa diagnosis using plasma sEV LAMB1 is shown in Figure 5B with area under curve (AUC) values of 0.9524 [95% confidence interval (CI) from 0.8769 to 1; *P*<0.0001] and 0.8643 (95% CI from 0.7282 to 1; *P* = 0.0001) compared to control and localised primary PCa, respectively. These data suggest that the diagnostic value with plasma sEV protein LAMB1 is higher than when using the serum total PSA value (AUC = 0.9381, 95% CI from 0.8472 to 1, *P*<0.0001, and AUC = 0.8262, 95% CI from 0.6797 to 0.9727, *P* = 0.0006, respectively).

Importantly, the combination of sEV protein LAMB1 and serum PSA was found to further improve the diagnostic value in the metastasis group compared to the control group (healthy and BPH participants) and localised primary PCa group up to AUC values 0.9905 (95% CI from 0.9657 to 1; *P* < 0.0001) and 0.9333 (95% CI from 0.8647 to 1; *P* < 0.0001), respectively. In addition, a distinct higher expression of sEV protein LAMB1 was also observed in the PCa group compared to the control group (*P* < 0.0001) (Figure 5A). ELISA results showed a significant difference in sEV protein LAMB1 expression between PCa and control groups, further demonstrating an improved diagnostic value of sEV LAMB1 in PCa with a higher individual AUC value at 0.9182 (95% CI from 0.8167 to 1; *P* < 0.0001) than serum PSA value at 0.8242 (95% CI from 0.7154 to 0.9331; *P* = 0.0002), or combined LAMB1 and PSA AUC value of 0.9348 (95% CI from 0.8495 to 1; *P* < 0.0001), (Figure 5B).

Similarly, urinary sEV protein Histone H4 was able to distinguish between low‐risk and high‐risk PCa patients (*P*<0.0001) (Figure 5C), and had higher diagnostic value (AUC = 0.9422, 95% CI from 0.8639 to 1; *P*<0.0001) in comparison to serum PSA for PCa stratification in a single PSA test (AUC = 0.7244) and in the combination of Histone H4 and serum PSA (AUC = 0.9733). When comparing the control, low‐risk PCa, high‐risk PCa, and mPCa groups, LAMB1 and Histone H4 were found to have the highest levels in mPCa plasma samples or high‐risk urine PCa samples, respectively (Figure 5D,E), indicating that ELISA results are consistent with PRM data (Figure 4).

In summary, these results suggest that sEV proteins LAMB1 in blood and Histone H4 in urine are superior to serum PSA in PCa diagnosis. More importantly, the combination of sEV protein LAMB1 and PSA has an improved diagnostic value for mPCa patients. In addition, the combination of sEV protein Histone H4 and PSA is better than any single test to distinguish between low‐ and high‐risk PCa patients for risk stratification.

### 2.6 Expression and Clinical Association of LAMB1 and Histone H4 Proteins in Human PCa Tissue

**Figure 6:**
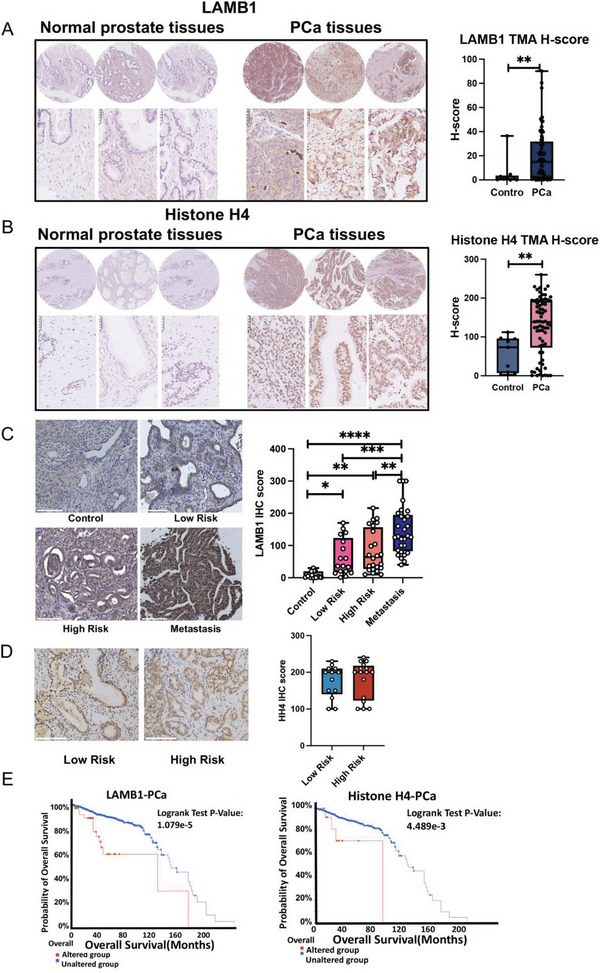
Clinical association of LAMB1 and Histone H4 in human PCa tissue and prognostic values in PCa patients. A, B) LAMB1 and Histone H4 protein expression in normal and PCa tissue were screened using a tissue microarray (TMA) (n = 9 for normal control and n = 70 for PCa tissue). Representative images showing a high expression of LAMB1 protein in PCa tissue compared to normal prostate tissue. Quantitative analysis indicates distinct expression of LAMB1 (*P* = 0.0094) and Histone H4 (*P* = 0.0045) in PCa tissues compared to normal prostate tissues. Brown colour indicates positive staining while blue colour indicates nuclear. Magnification 40x, scale bar = 50 mm. C) Expression of LAMB1 protein in different tissues with different PCa staging by IHC staining (n = 18, 15, 24, and 29 for normal control, low‐risk, high‐risk, and metastasis groups, respectively). Representative images showing high expression of LAMB1 in different PCa stages compared to normal prostate tissue. Quantitative analysis indicates metastasis distinct to high‐risk (*P* = 0.0014), low‐risk (*P* = 0.0009), control (*P* < 0.0001); high risk distinct to control (*P* = 0.0012); low‐risk distinct to control (*P* = 0.0254). Brown colour indicates positive staining while blue colour indicates nuclear. Magnification 20x. D) Representative images showing high Histone H4 expression in both low‐ (n = 13) and high‐risk (n = 16) stages of PCa tissue. Quantitative analysis indicates no significant difference was observed between low‐risk and high‐risk stages of PCa tissue (*P* > 0.05). Brown colour indicates positive staining while blue colour indicates nuclear. Magnification 20x. E) The association of intratumoural LAMB1 and Histone H4 protein expression with overall survival status in the patient cohort (n = 1476) from the cBioPortal dataset was analysed using the Kaplan‐Meier method and log‐rank test (*P* < 0.01).

To further investigate the association of sEV proteins with human PCa in clinics, human tissue samples were profiled using immunohistochemistry (IHC) staining. Evaluation of both LAMB1 and Histone H4 proteins in PCa and normal prostate tissues using commercial TMA found that both markers (*P* = 0.0094 and 0.0045, respectively) have increased expression in PCa tissue compared to normal tissue (**Figure** **6**A,B), indicating that relative expression of sEV proteins LAMB1 and Histone H4 in PCa plasma and urine is aligned with PCa tissue and that possibly sEV LAMB1 in blood and Histone H4 in urine are secreted by PCa cells.

As tissue samples with different stages of PCa using commercial TMAs were not available, PCa tissues obtained during surgery and stored in our tissue bank were evaluated for expression of the two markers. The samples were categorised as either low‐risk, high‐risk, or mPCa. A distinct expression pattern of LAMB1 protein was observed in the metastasis group compared to high‐risk (*P* = 0.0014), low‐risk (*P* = 0.0009), and control (*P*<0.0001) groups; as well as in high‐risk compared to control group (*P* = 0.0012) and low‐risk compared to control group (*P* = 0.0254). The expression of plasma sEV LAMB1 within different groups of PCa was consistent with the tissue expression of LAMB1 protein in the matched patient groups, suggesting a possible biological link between the two sample sources. However, there was no significant difference in the expression of Histone H4 between low‐risk and high‐risk of PCa tissue (*P* > 0.05) (Figure 6D), which is not consistent with the expression pattern of urine sEV Histone H4, indicating other factors may affect sEV Histone H4 expression.

The clinical significance of LAMB1 and Histone H4 proteins was also investigated using PCa patient data from the cBioPortal dataset (n = 1476) (Figure 6E). The data indicated that a high intratumoral expression of LAMB1 and Histone H4 was associated with a low overall survival rate in PCa patients with poor prognosis, further suggesting that both of these sEV proteins hold potential in PCa diagnosis, prognosis, and therapeutics.

## 3 Discussion

The incidence of PCa is rising worldwide.^[^ ^17^ ^]^ The treatment choice for individual patient is different among these males. mPCa is one of the most common causes of patient death. According to the US Surveillance, Epidemiology, and End Results database, PCa kills more than 350,000 lives worldwide every year and the 5‐year survival rate of mPCa is only 30.5%.^[^ ^18^ ^]^ Risk stratification is the major strategy for determining the most appropriate treatment option for an individual. The current approach for risk stratification is mainly based on historical evidence such as TNM (tumour, node, and metastasis) staging and Gleason score. However, this system is not originally verified against PCa mortality. Furthermore, no existing risk stratification approach has been established initially in an unscreened population, which accounts for the vast majority of men suffering with PCa worldwide. Current tools used in PCa diagnosis and risk progression stratification are not accurate and sufficient for making clinical decisions for patients’ therapy choice. Therefore, there is an urgent need for new tools to improve PCa diagnosis and risk stratification.

EVs from liquid biopsy has shown great potential for early cancer detection with the rapid feedback, minimal invasiveness, and a source of homogeneously circulating biomarkers that can be monitored in a real‐time. Theodoraki et al. reported circulating sEVs carrying PD‐L1 were associated with disease progression in head and neck squamous cell carcinoma patients whereas soluble PD‐L1 in plasma was not.^[^ ^19^ ^]^ Similarly, evidence has shown that serum sEV protein GPC1 from pancreatic cancer patients has absolute specificity and sensitivity to distinguish healthy controls, demonstrating that it is a reliable biomarker for the detection of early‐stage of pancreatic cancer.^[^ ^20^ ^]^ These data support the concept that sEV protein biomarkers hold potential in cancer early diagnosis and progression stratification.

Previously, we showed that the current existing clinical diagnostic marker‐PSA widely studied may be insufficient to be directly adapted to sEVs for PCa diagnosis and risk stratifications.^[^ ^12^ ^]^ Therefore, we performed quantified proteomic analysis to discover novel sEV‐derived PCa protein biomarkers. Unlike from body fluids and tissues, sEV biomarkers derived from PCa cell lines contain biological cargo information directly related to PCa, which are not affected by biological regulatory factors such as the immune response or tumour microenvironment (TME), hence have the advantages of high specificity and purity of PCa‐related sEVs. Human PCa cell lines from differing origin [PC3 (bone metastasis), DU145 (brain metastasis), LNCaP (lymph node metastasis), and 22Rv1 (primary tumour)] express various sEV protein cargos compared to the normal prostate epithelial cell (RWPE‐1). In contrast to EVs purified from complex sources such as bodily fluids and tissues, EVs from PCa cell lines contain biologically relevant information directly associated with PCa. They are not influenced by biological factors such as the TME, immune system, hypoxia or therapy, demonstrating advantages including high specificity and purity.

In this study, proteomic analysis of sEVs from 4 distinct PCa cell lines was performed. Metastatic cell lines with different gene mutations may have different sEV secretions.^[^ ^21^ ^]^ For example, aside from some common mutations in RCF2, TP53BP1, and AR genes, the GTF2H3 and SMARCAL1 mutations in PC3 cells, the BRCA1 and IPMK mutations in DU145 cells, the ABL1 and PTEN mutations in LNCaP cells, and the EME1 and HELLS mutations in 22Rv1 cells, could potentially affect sEV secretion.^[^ ^22^ ^]^ However, sEV isolation in these cell lines were performed under the same culture conditions for this study. In addition, we used the same control criteria for the comparison of different PCa cell lines to minimise differences to the best of our effort. More importantly, our results have been validated in human plasma and urine clinical samples to avoid these influences. Subsequently, we discovered primary and metastatic PCa cell‐type specific sEV proteins using label‐free MS proteomics. Our findings indicate sEV cargos are cell type‐dependent, demonstrating the fluctuation of sEV protein levels as the development of PCa. Our verification study indicates sEV proteins LAMB1 and Histone H4 that were verified in PCa plasma and urine EVs urine samples also matched the expression on human PCa tissues, suggesting that the potential sEV protein markers identified in PCa cell lines are PCa specific and are worth for further validation.

Several sEV proteins in primary PCa and mPCa cells were identified using label‐free MS proteomics in the current study. Some of them, such as RTN4, CLTC, CD151, LAMB1, and NRP1, have been reported to be associated with cancer progression.^[^ ^23^ ^]^ Others, such as LARS1 and FLNC, were reported to be associated with cancer migration.^[^ ^24^ ^]^ Histone H4, ABI3BP, RAN, and FLNA were found to be associated with cancer development.^[^ ^25^ ^]^ PXDN, URP2, DNAJA1, and MYH9 were demonstrated to be associated with cancer metastasis.^[^ ^26^ ^]^ Histone H3.1, AHCY, and ADK were demonstrated to be associated with cancer regulation.^[^ ^27^ ^]^ Furthermore, LAMB2 and SPON2 were shown to be associated with cancer biomarkers and the microenvironment, respectively (Table S3, Supporting Information).^[^ ^28^ ^]^ However, to the best of our knowledge, a comprehensive proteomic analysis of mPCa cells‐derived sEVs for diagnosis and risk stratification has not been reported so far.

In recent years, MS‐based quantitative proteomics has been widely used for cancer biomarker discovery. For example, Mariscal et al. identified three PCa‐specific palmitoyl‐proteins with label‐free proteomics in both sEV and lEV subpopulations.^[^ ^29^ ^]^ Using label‐free phosphoproteomics, Chen et al. discovered 144 phosphoproteins in plasma sEVs that are considerably higher in breast cancer patients compared to healthy controls,^[^ ^30^ ^]^ suggesting the usefulness of MS‐based proteomics in cancer biomarker discovery. Label‐free quantitative proteomic strategies are suitable for discovering biomarkers with larger protein expression changes between different biological states.^[^ ^31^ ^]^

In this study, absolute quantitation by PRM acquisition was used to show that expression sEV proteins LAMB1 in plasma and Histone H4 in urine matched the trends observed in cell‐derived sEVs. Furthermore, PRM data showed that plasma sEV LAMB1 expression can significantly distinguish mPCa from high‐risk PCa, and sEV protein Histone H4 expression in urine can distinguish PCa patients with high‐risk versus low‐risk. The treatment options for different stages of PCa vary depending on whether low‐risk, high‐risk, and mPCa can be differentiated. In the case of low‐risk PCa, treatment options typically include surgical removal or strict surveillance. On the other hand, high‐risk PCa is commonly managed using hormone therapy and chemotherapy. For mPCa, the recommended treatment approach involves chemotherapy and radiotherapy. Therefore, biomarker discovery is of critical importance for the clinical management of PCa.

Laminins, a family of extracellular matrix glycoproteins, are the major noncollagenous constituent of basement membranes. They are involved in various biological processes such as cell adhesion, differentiation, migration, signalling, neurite outgrowth, and metastasis. They consistent of three different chains (alpha, beta, and gamma) allowing different combinations and giving rise to different laminin isoforms. The specific functions of the different chains and isoforms are not fully understood, but they are believed to have diverse roles in different tissues. Laminin subunit beta‐1, or LAMB1, is one of the three chains that constitute laminin 1. One specific domain of LAMB1 has been identified as important for cell attachment, chemotaxis, and binding to the laminin receptor, and it has shown a clear association with metastasis.^[^ ^32^ ^]^ LAMB1 is involved in integrin and ERK signalling. In this study, we first find LAMB1 plays a significant role in cancer cell communication and mediating PCa metastasis through sEVs. The role of LAMB1 in basement membranes and tumour‐derived sEVs may be related to communication between PCa cells and the TME. Combining the previously reported role of LAMB1 with our current results, we hypothesise that sEV LAMB1 protein may participate in pre‐metastatic niche formation in a vesicle‐mediated manner, but further investigation is required to validate this statement.

Histone H4 is one of the major histone proteins participating in the structure of chromatin in eukaryotic cells. Numerous studies have shown that Histone H4 and its post‐translational modifications (PTM) are highly associated with cancer development. Carrer et al. reported that Histone H4 acetylation levels were increased in pancreatic ductal adenocarcinomas harbouring Kras mutations prior to the premalignant lesion change, and acetyl‐CoA metabolism contributed to metabolic or epigenetic modification promoted pancreatic tumourigenesis.^[^ ^25a^ ^]^ Furthermore, Yuan et al. also reported that Histone H4 lysine crotonylation participated in glioblastoma stem cell lysine catabolism reprograming leading to enhanced tumour growth and TME changes.^[^ ^33^ ^]^ Another interesting study from Shah et al. reported extracellular Histone H4 was associated with elevated microparticle production in trauma haemorrhage patients.^[^ ^34^ ^]^ Several studies have shown that histones are present in sEV populations.^[^ ^35^ ^]^ Together, these studies suggest that Histone H4 could be associated with extracellular transport and plays a critical role in cancer progression.

Our study found that sEV protein Histone H4 expression in urine samples from PCa patients has higher AUC values than the serum PSA test in differentiating high‐ and low‐risk PCa patients or control subjects, indicating that sEV Histone H4 proteins could be used to distinguish high‐ and low‐risk PCa patients and to facilitate PCa stratification. Currently, EV‐associated DNA research remains elusive. Our study showing the importance of DNA‐associated protein in EVs may provide useful insight into the way in which this complicated problem may be solved in the future.

Previous studies have shown that LAMB1 protein expression was increased in gastric cancer tissues and associated with poor overall survival, first progression, and post‐progression survival of gastric cancer patients.^[^ ^36^ ^]^ In PCa, LAMB1 expression was reported to be linked with cell motility and invasion into the surrounding extracellular matrix but not significantly associated with survival.^[^ ^37^ ^]^ Studies on Histone H4 and its PTM variants demonstrated that it was associated with cell proliferation, migration, and regulation of PCa progression, but survival results were inconsistent.^[^ ^38^ ^]^ Although the data from the cBioPortal dataset indicated survival advantages for both LAMB1 and Histone H4 markers, the results may be limited by different variables and event numbers in the disease group.

One of the limitations of MS analysis is that low‐abundant peptides are masked by the presence of high‐abundant peptides. Therefore, this may suppress the appearance of low abundant EV markers that may overlap with all five cell line‐derived sEV populations. In addition, the use of a limited number of cell lines, along with their specific biological properties, may not encompass the entire profile of PCa. This study has only focused on a subpopulation of EVs (sEVs) from PCa cells and other EV subpopulations such as lEVs may contain additional PCa EV biomarkers which are beyond the scope of this investigation.

In conclusion, we have identified 20 novel sEV protein biomarkers from PCa cell lines using label‐free proteomics and validated two potential biomarkers in human plasma and urine samples from patients at different stages of PCa using targeted MS, WB and ELISA. Our findings indicate the sEV protein LAMB1 can differentiate mPCa patients from high‐risk PCa patients while the sEV protein Histone H4 can distinguish patients with high‐ and low‐risk PCa. However, a large set of clinical samples are needed to further verify the findings in multi‐centres and to establish real clinical values. Taken together, our findings support the potential of sEV proteins LAMB1 and Histone H4 in clinical applications and can provide valuable information for PCa detection, risk progression stratification, and best treatment strategy options.

## 4 Experimental Section

### CCM Preparation

PCa cell lines including PC3, DU145, LNCaP, and 22Rv1 were purchased from the American Type Culture Collection (ATCC, VA, USA) and cultured in RPMI 1640 medium at 37°C. RWPE‐1 was purchased from FuHeng Biology (Shanghai, China) and cultured in a Defined K‐SFM medium. Cells were cultured until 60–70% confluency followed by a gentle wash with phosphate‐buffered saline (PBS) twice and incubated in a fresh exosome‐depleted medium for 48 h. All cell lines were confirmed to be free of mycoplasma contamination and validated with a cell line authentication service using short tandem repeat markers.

### Pre‐Purification for Cell Culture Supernatants

Cell medium was centrifuged at 300 × g for 5 min at room temperature (RT) to remove live cells. The supernatant was collected and centrifuged at 2,000 × g for 20 min at 4 °C to remove dead cells, followed by 10,000 × g for 30 min to remove aggregates of biopolymers, cellular debris, and other structures with a buoyant density higher than that of sEVs. The supernatant was collected and filtered with a 0.22 µm pore filter.

### Isolation of sEVs from Cell Media

sEVs from PCa cell media were isolated using the Total Exosome Isolation kit (Life Technologies, Cat No. 4478359) following the manufacturer's instructions. Briefly, the cell medium was incubated with the reagent overnight (o/n) with a ratio of 2:1 v/v at 4 °C. After incubation, the cell medium was centrifuged at 10,000 × g for 1 h at 4 °C. All supernatant was carefully removed without disturbing the pellet. Filtered PBS or an appropriate solution was used to resuspend the pellet. sEV samples were aliquoted and stored at −80 °C until further use.

### Clinical Samples

Peripheral blood (n = 107), first void urine (n = 106), and tissue samples (n = 92) were collected from histologically confirmed PCa patients with or without metastasis or no‐cancer control men 40 years of age or older under informed consent and with ethical approval from Ningbo First Hospital, Ningbo University (Approval no:2021‐R106). Enrolled clinical subjects were grouped according to pathologic diagnosis including localised PCa group, metastasis group, and control group. Localised groups were further stratified to low‐, intermediate‐, high‐, and very high‐risk groups according to NCCN guidelines.^[^ ^39^ ^]^ Taking statistical analysis into consideration and a balanced number of patient groups, very high‐ and high‐risk patients were grouped as high‐risk; and intermediate‐ and low‐risk patients as low‐risk.

During the validation phase, two individual cohorts were enrolled. Validation cohort 1 included plasma samples (12 non‐cancer control, 12 low‐risk, 12 high‐risk, 11 metastatic) and urine samples (12 non‐cancer control, 11 low‐risk, 12 high‐risk, 11 metastatic) from 50 participants. Validation cohort 2 included plasma samples (15 non‐cancer control, 15 low‐risk, 15 high‐risk, 14 metastatic) and urine samples (15 non‐cancer control, 15 low‐risk, 15 high‐risk, 14 metastatic) from 89 participants. Tissue samples from 77 participants (4 non‐cancer control, 15 low‐risk, 24 high‐risk, 34 metastatic), and all plasma and urine samples were collected from Ningbo First Hospital (Zhejiang, China) between 2021–2023. All biofluid samples were obtained from fasting individuals prior to receiving any therapeutic intervention. Men with infectious disease, on medication that could influence serum PSA levels, a history of other cancers, or invasive treatment within 3 months, were excluded. Specific clinical patient information is shown in Table S2 (Supporting Information).

The Declaration of Helsinki was followed in the conduct of the study. This investigation was carried out in accordance with the Standards for Reporting Diagnostic Accuracy recommendations. Written permission was given voluntarily by every patient.

### Preparation of Human Plasma

Whole blood from PCa patients and age‐matched control subjects was collected using EDTA‐treated tubes (BD bioscience, Jiangsu, China). All blood samples were processed within 2 h after collection from patients and controls. Whole blood samples were centrifuged for 15 min at 3000 rpm to collect raw plasma. Collected plasma was transferred to a clean tube immediately and centrifuged at 2000 × g for 20 min at 4 °C to remove cell debris. Supernatants were transferred to a clean tube and centrifuged at 10,000 × g for 30 min at 4 °C to remove large particles and aggregates. The cleared plasma was aliquoted and stored at −80 °C.

### Preparation of Human Urine

First‐morning urine from PCa patients and age‐matched control subjects was collected using sanitised urine containers. All urine samples were processed within 2 h after collection from patients. Whole urine samples were centrifuged for 10 min at 4,000 rpm to remove cells and bacteria. Collected supernatants were transferred to a clean tube immediately and centrifuged at 2,000 × g for 10 min at 4 °C to remove cell debris or stones. Supernatants were transferred to a clean tube and centrifuged at 10,000 × g for 30 min at 4 °C to remove large particles and aggregates. The cleared urine was aliquoted and stored at −80 °C.

### Ultracentrifugation

Fresh or thawed cell culture supernatant, plasma, or urine samples were diluted with cold PBS and centrifuged with a Beckman Coulter Type 70 Ti fixed angle rotor (adjusted k‐factor 131, maximal acceleration, maximal deceleration) at 110,000 × g for 2 h at 4 °C. After the first spin, sEV pellets were resuspended in cold PBS followed by a second spin using a Beckman SW41Ti rotor at 110,000 × g for 2 h at 4 °C. The final pellet was resuspended in a final volume of 100–200 µLwith fresh filtered cold PBS followed by protein quantification.

### WB

Total protein was extracted with RIPA lysis buffer (Cell Biolabs) containing 1 × Halt protease and phosphatase inhibitor cocktail (Thermo Scientific, USA), while sEV protein was further lysed by ultrasound in an iced water bath for 15 s for 4 rounds and put on ice for 10 s in between each round. Protein was quantified with the BCA Protein Assay Kit (Thermo Fisher Scientific, China). Equal amounts of protein (1.7–20 µg) from the same type of samples were separated on a 4–12% Bis‐Tris protein gel (Bio‐Rad, USA) and blotted onto 0.22 µm polyvinylidene fluoride membranes (Millipore/Merck). The membrane was blocked with 5% BSA in Tris Buffered Saline with Tween‐20 (TBST) buffer for 1 h at RT, followed by incubation with primary antibodies o/n at 4 °C. Antibodies were diluted according to the manufacturer's recommendation. After washing 4 × 5 min with TBST, the membrane was incubated with HRP‐conjugated secondary antibody (Thermo Fisher Scientific) at RT for 1 h, followed by another 4 × 5 min washing with TBST. Immunoblot bands were detected with enhanced chemiluminescence WB substrate (SuperSignal West Dura, Thermo Fisher Scientific), and imaged using a ChemiDoc Imaging System (Bio‐Rad). Antibodies were obtained from different sources with detailed information listed in **Table** **2**.

**Table 2:** Antibodies used for WB and IHC.

Antibody	Source	Application	Catalogue number
CD81	Abcam (China)	WB	ab79559
Syntenin‐1	Abcam (China)	WB	ab133267
Flotillin‐1	Abcam (China)	WB	ab133497
Calnexin	Abcam (China)	WB	ab133615
LAMB1	Affinity Biosciences (China)	WB, IHC	DF3618
Histone H4	Proteintech (China)	WB, IHC	16047‐1‐AP
PXDN	MERCK (USA)	WB	ABS1675
DNAJA1	Abcam (China)	WB	ab126774
URP2	Abcam (China)	WB	ab173416

### NTA

NTA (NanoSight NS300, Malvern Pananalytical, UK) was performed using our previously published method.^[^ ^12^ ^]^ Briefly, sEV samples were diluted to a suitable range with particle concentrations between 108–109 particles mL^−1^. For each sample, five 60 s videos were captured at camera level 13 and threshold 5 (NTA 3.4). The original particle concentrations from the isolates were then calculated based on the measured concentrations and the dilution factor.

### TEM

sEV samples were assessed by TEM for characterisation. Briefly, 10 µL of each sample was adsorbed to an ultra‐thin carbon‐coated 400 mesh copper grid and incubated for 5 min at RT. Then, grids were negatively stained with filtered 2% uranyl acetate and then the excessive stain was removed. Next, the grids were completely dried in darkness and imaged by TEM operating at 80 kV.

### Proteomic Analysis of sEVs from CCM by LC‐MS/MS

For protein extraction of sEVs from CCM, SDT (4% SDS, 1 mM DTT, 100 mM Tris‐HCl, pH 7.6) buffer was used for sample lysis and protein extraction. The DTT, SDS, and other low molecular weight components were eliminated by ultrafiltration (Millipore, 10 kD) using UA buffer (8 M urea, 150 mm Tris‐HCl, pH 8.0). The samples were then treated with 100 µL iodoacetamide (IAA) solution (100 mM IAA in UA buffer) in darkness for 30 min to block reduced cysteine residues, followed by three times wash with UA buffer and NH_4_HCO_3_ buffer (25 mm), respectively. The samples were digested with trypsin o/n at 37 °C. The digested peptides of each sample were desalted on C18 Spin Tips (Thermo Scientific), concentrated by SpeedVac centrifugal evaporator, and then resolved in 0.1% formic acid.

Protein tryptic digests were analysed by LC‐MS/MS using a hybrid quadrupole‐Orbitrap tandem mass spectrometer (Q‐Exactive, Thermo Fisher Scientific, USA) that was coupled to an EASY‐nLC system. MS data was acquired using a data‐dependent acquisition mode in the m/z range from 300–1800 for higher‐energy collision dissociation (HCD) fragmentation. The resolution was set to 17,500 at m/z 200, and the isolation width was 2 m z^−1^ for HCD spectra.

For identification and quantitation analysis, the raw MS data in each sample were combined and searched using MaxQuant 1.5.3.17 software (Max Planck Institute of Biochemistry, Germany). The peptides and proteins were identified using the Universal Protein Resource database (Uniprot) and following parameter set: (1) taxonomy, homo sapiens (human); (2) enzyme, Trypsin; (3) maximum missed cleavages, 2; (4) fixed modifications, carbamidomethyl (C); (5) variable modifications, oxidation (M); (6) main search, 6 ppm; (7) first search, 20 ppm; (8) ms/ms tolerance, 20 ppm. The false discovery rate (FDR) at the peptide and protein levels was set ≤ 0.01. Razor and unique peptides were used for protein quantification. Peptide abundances were compared by one‐way ANOVA.

### PRM Analysis of sEVs from Human Plasma and Urine Samples

Targeted proteomics (PRM) was performed to validate the selected sEV proteins from human plasma and urine samples on a Q Exactive TM HF MS (Thermo Fisher Scientific) system coupled with an UltiMate TM 3000 RSLCnano system (Thermo Fisher Scientific). Each selected precursor had no modification other than carbamidomethyl (C) and no missed cleavages, with the peptide length between 7 and 25 residues using Skyline software (Version 21.1). For retention time calibration, 10 common internal retention time standard peptides were added.

≈ 30 µg of sEV proteins were prepared for LC‐MS/MS analysis by Workflow A of pressure cycling technology (PCT)‐assisted lysis and digestion protocol described previously.^[^ ^40^ ^]^ Specifically, sEV lysates were digested with 1 µg trypsin (Hualishi Technology, Beijing, China) and 0.25 µg LysC (Hualishi Technology, Beijing, China). Peptides were cleaned up using SOLAμ HRP (Thermo Scientific). The processed peptides were dissolved in buffer A and separated at 300 nL min^−1^ along with a 30 min 5–30% buffer B linear LC gradient (buffer A: 2% ACN, 0.1% formic acid; buffer B: 98% ACN, 0.1% formic acid) using an analytical column (75 µm × 150 mm, 1.9 µm, 120 Å C18 particles). The acquisition cycle consisted of a full scan and 15 scheduled PRM MS/MS scans in a ± 2.5 min retention time window. A full MS scan was acquired at a resolution of 60,000, with an automatic gain control (AGC) goal value of 3 × 10^6^ and a maximum injection time (IT) of 55 ms. Target ions were transported to MS/MS in the HCD cell (1.6 m/z isolation width, 27% normalised collision energy). MS/MS spectra were acquired at resolution 30,000 with an AGC target value of 2 × 10^5^ and a maximum IT of 100 ms.

### TMA and IHC Analysis

TMA slides (M079Pr01) with human prostate tissue including 69 PCa and 9 normal controls were purchased from Bioaitech (Xi'An, China). Since commercially available TMAs do not include metastatic tissues, additional tissues including metastases were obtained from the own tissue archive at Ningbo First Hospital (Human ethics approval number: 2021‐R106). IHC staining and analysis were performed on both TMA and tissue collected from Ningbo First Hospital. Briefly, tissue sections were deparaffinised in xylene and rehydrated in graded ethanol (95%, 75%, 50%, and distilled water). Slides were cleaned and immersed in 95 °C 0.01 M citrate buffer for 30 min to recover antigen and then sealed with goat serum at RT for 1 h before incubation with rabbit primary polyclonal antibodies against LAMB1 (Affinity Biosciences, Cat. No.DF3618) and Histone H4 (Proteintech, Cat. No. 16047‐1‐AP) diluted at 1:400 and 1:2500, respectively, at 4°C o/n. The slides were developed using liquid diaminobenzidine (Agilent, CA, USA) on the second day after 1 h of incubation with a secondary antibody at RT. A light microscope (Leica, Wetzlar, Germany) was used to measure staining intensity. Both intensity and histochemistry score (H‐score) were used to evaluate IHC results.^[^ ^41^ ^]^ The intensity of IHC staining was defined as follows: 0 = none, 1 = weak, 2 = moderate, and 3 = strong. H‐scores ranged from 0 to 300. The results were scored from two independent observers (BRP and WSY). Specific TMA patient information is shown in Table S3 (Supporting Information).

### Statistical Analysis

Data analysis was performed using GraphPad Prism version 8 (GraphPad Software, San Diego, CA, USA). Data are reported as means and standard error of the mean. ANOVA analysis was performed for parametric tests with the Tukey method as a post‐hoc test. The Mann‐Whitney test was used for nonparametric tests with a two‐tailed *P* value. The *P* value of <0.05 was used to evaluate the significance of the data. AUC, ROC, and other metrics were used to evaluate the diagnostic efficiency. Kaplan–Meier survival curves were generated with the log‐rank test.

## Conflict of Interest

The authors declare no conflict of interests

## Author Contributions

L.Y., P.B.R., and J.J.H. conceived the idea of the study. PBR performed the experiments, article writing, and created the figures and tables. L.Y. guided the preparation of this manuscript. Y.L., D.X., and G.T.N. provided support for targeted M.S. Z.C., P.B.R., W.Q., C.H.T., L.Z.H., H.M., G.J., Y.Z.J., C.Y.Z., and W.S.Y. performed the sample collection and data acquisition. MD, BJ, GTN, JJH, and LY conducted paper revisions. All authors read and approved the final manuscript.

## Supporting information

Supporting Information

Supporting Information

## Data Availability

The data that support the findings of this study are available on request from the corresponding author. The data are not publicly available due to privacy or ethical restrictions.
