# Supplementary material for: Proteomic Identification of Small Extracellular Vesicle Proteins LAMB1 and Histone H4 for Prostate Cancer Diagnosis and Risk Stratification
Source: Adv Sci (Weinh). 2024 Apr 8;11(23):2402509. doi: 10.1002/advs.202402509 (PMC11187897; doi:10.1002/advs.202402509)
Supplement: Supplementary file 1 — Supporting Information [file ADVS-11-2402509-s001.pdf]

ADVANCED SCIENCE
Open Access

Supporting Information

for *Adv. Sci.*, DOI 10.1002/advs.202402509

Proteomic Identification of Small Extracellular Vesicle Proteins LAMB1 and Histone H4 for Prostate Cancer Diagnosis and Risk Stratification

Bairen Pang, Qi Wang, Haotian Chen, Zhihan Liu, Meng Han, Jie Gong, Liang Yue, Xuan Ding, Suying Wang, Zejun Yan, Yingzhi Chen, David Malouf, Joseph Bucci, Tiannan Guo, Cheng Zhou, Junhui Jiang* and Yong Li**

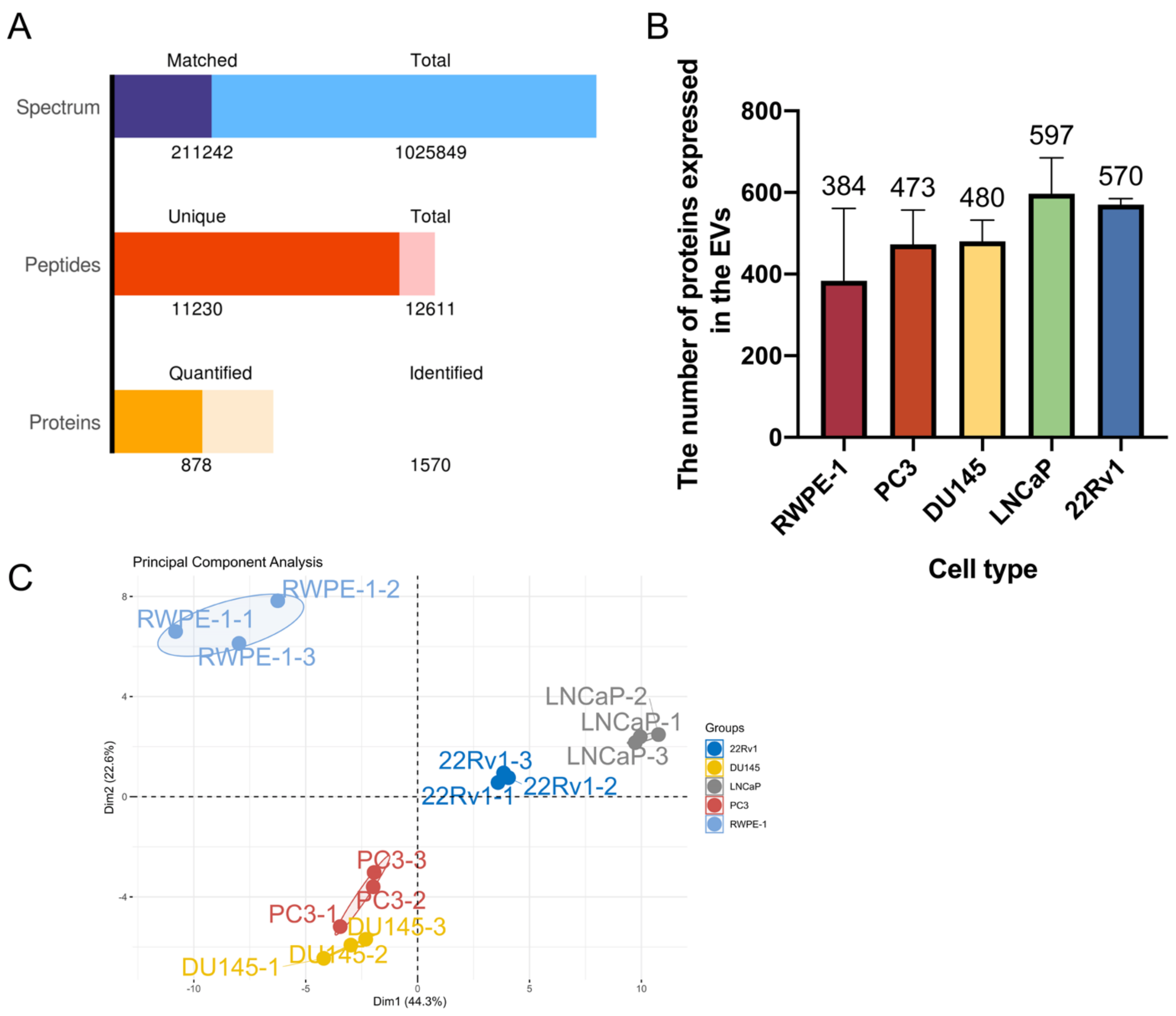

Figure S1. Characterisation of sEV proteins from different PCa cell lines and a normal prostate epithelial cell line using a label-free proteomic method.

(**A**) Identification and quantification results of proteomics with total proteins identified in all cell lines. (**B**) Number of sEV proteins expressed in five different cell lines. (**C**) PCA visualisation of the sEV protein difference from five different cell lines.

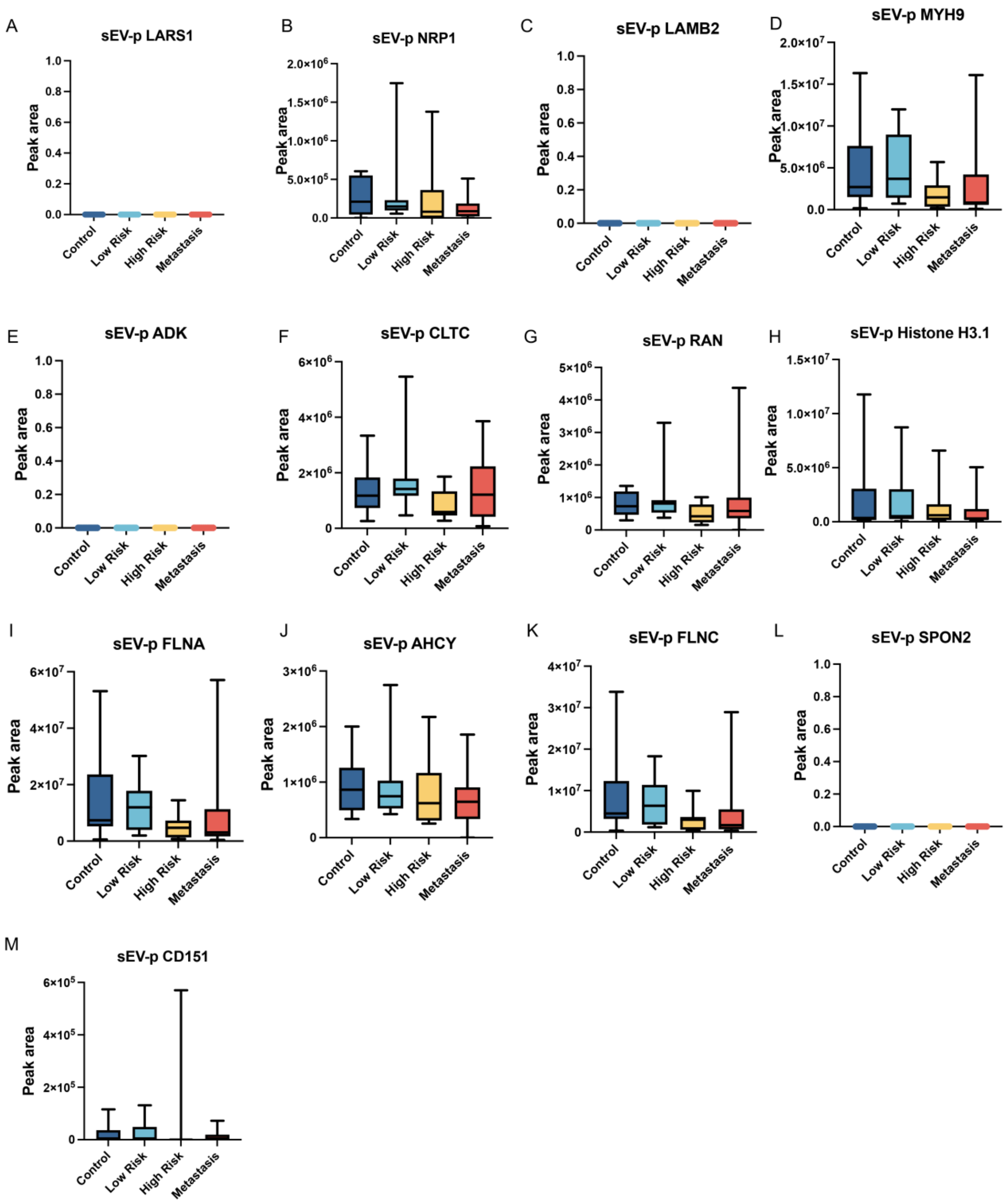

Figure S2. PRM characterisation of plasma derived sEV proteins from different clinical groups. (**A**) Plasma derived sEV protein LARS1 level in control, low-risk, high-risk and metastasis group people. (**B**) Plasma derived sEV protein NRP1 level in control, low-risk, high-risk and metastasis

group people. (**C**) Plasma derived sEV protein LAMB2 level in control, low-risk, high-risk and metastasis group people. (**D**) Plasma derived sEV protein HYH9 level in control, low-risk, high-risk and metastasis group people. (**E**) Plasma derived sEV protein ADK level in control, low-risk, high-risk and metastasis group people. (**F**) Plasma derived sEV protein CLTC level in control, low-risk, high-risk and metastasis group people. (**G**) Plasma derived sEV protein RAN level in control, low-risk, high-risk and metastasis group people. (**H**) Plasma derived sEV protein Histone H3.1 level in control, low-risk, high-risk and metastasis group people. (**I**) Plasma derived sEV protein FLNA level in control, low-risk, high-risk and metastasis group people. (**J**) Plasma derived sEV protein AHCY level in control, low-risk, high-risk and metastasis group people. (**K**) Plasma derived sEV protein FLNC level in control, low-risk, high-risk and metastasis group people. (**L**) Plasma derived sEV protein SPON2 level in control, low-risk, high-risk and metastasis group people. (**M**) Plasma derived sEV protein CD151 level in control, low-risk, high-risk and metastasis group people.

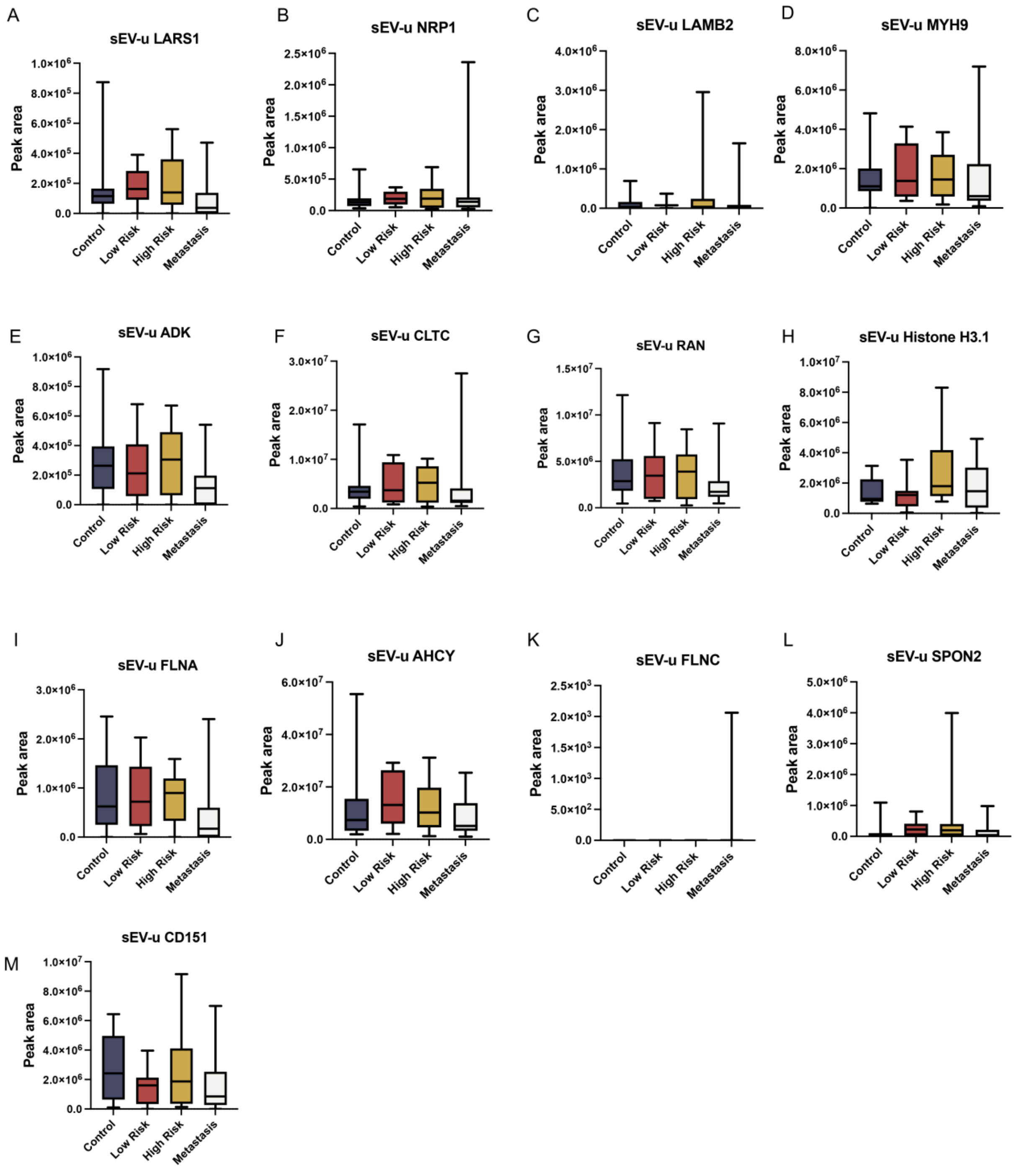

Figure S3. PRM characterisation of urine derived sEV proteins from different clinical groups. (**A**) Urine derived sEV protein LARS1 level in control, low-risk, high-risk and metastasis group people. (**B**) Urine derived sEV protein NRP1 level in control, low-risk, high-risk and metastasis group people. (**C**) Urine derived sEV protein LAMB2 level in control, low-risk, high-risk and metastasis

group people. (**D**) Urine derived sEV protein HYH9 level in control, low-risk, high-risk and metastasis group people. (**E**) Urine derived sEV protein ADK level in control, low-risk, high-risk and metastasis group people. (**F**) Urine derived sEV protein CLTC level in control, low-risk, high-risk and metastasis group people. (**G**) Urine derived sEV protein RAN level in control, low-risk, high-risk and metastasis group people. (**H**) Urine derived sEV protein Histone H3.1 level in control, low-risk, high-risk and metastasis group people. (**I**) Urine derived sEV protein FLNA level in control, low-risk, high-risk and metastasis group people. (**J**) Urine derived sEV protein AHCY level in control, low-risk, high-risk and metastasis group people. (**K**) Urine derived sEV protein FLNC level in control, low-risk, high-risk and metastasis group people. (**L**) Urine derived sEV protein SPON2 level in control, low-risk, high-risk and metastasis group people. (**M**) Urine derived sEV protein CD151 level in control, low-risk, high-risk and metastasis group people.

Table S1. Patients' clinic grouping.

**Control group**	Age, sex matched subjects (including BPH),without cancer history
**Low-risk PCa group**	PSA ≤20 ng/mL, Gleason score≤7， TNM: T1 to T2c
**High-risk PCa group**	PSA >20 ng/mL or Gleason score>7 or TNM: T3a to T4
**Metastatic PCa group**	Regional or distant metastasis before therapy

Table S2. Clinical Patient information.

Characteristics	Patient information			
Group	Control	Low Risk	High Risk	Metastasis
Age (years)				
Average	66	68	70	70
(range)	43-81	55-78	53-81	54-88
PSA (ng/mL)				
Average	8	9	34	200
Range	0.15-23.91	0.2-19.64	5.78-154	0.66-1520
Gleason score				
Average	/	7	8	8
Range	/	6-7	7-10	7-10
Tumour stages, n (%)				
T1-T2a	/	7(4.7%)	/	/
T2b-T2c	/	32(21.3%)	12(8%)	1(0.7%)
T3a-T4	/		45(30%)	53(35.3%)
Nodal stage, n (%)				
N0	/	39(26%)	57(38%)	21(14%)
N1	/	/	/	33(22%)
Metastatic stages, n (%)				
M0	/	39(26%)	57(38%)	22(14.7%)
M1-M1c	/	/	/	32(21.3%)

Table S3. Cancer association of twenty discovered sEV proteins from literatures.

**Protein name**	**Cancer association from previous study**	**Cancer type**	**Reference**
RTN4	Cancer progression	Lung, breast, cervical, kidney, and ovarian cancers	[23a]
LARS1	Cancer migration	Lung cancer	[24a]
LAMB2	Cancer biomarker	Parathyroid Carcinoma	[28a]
PXDN	Cancer metastasis	Oral squamous cell carcinoma	[26a]
NRP1	Cancer progression	Prostate cancer	[23e]
LAMB1	Cancer progression	Hepatocellular carcinoma	[23d]
URP2	Cancer metastasis	Breast cancer	[26b]
ADK	Cancer regulation	Breast, prostate, liver, cervical cancer, glioma	[27c]
DNAJA1	Cancer metastasis	Head and neck squamous cell carcinoma	[26c]
AHCY	Cancer regulation	Breast cancer	[27b]
MYH9	Cancer metastasis	Gastric cancer	[26d]
Histone H3.1	Cancer regulation	Lung cancer	[27a]
CLTC	Cancer progression	Osteosarcoma	[23b]
Histone H4	Cancer development	Pancreatic cancer	[25a]
RAN	Cancer development	Ovarian cancer	[25c]
FLNA	Cancer development	Lung cancer	[25d]
CD151	Cancer progression	Lung cancer	[23c]
ABI3BP	Cancer development	Gallbladder cancer	[25b]
FLNC	Cancer migration	Lung cancer	[24b]
SPON2	Cancer microenvironment	Hepatocellular carcinoma	[28b]
